# Risk Factors Associated With Social Media Addiction: An Exploratory Study

**DOI:** 10.3389/fpsyg.2022.837766

**Published:** 2022-04-14

**Authors:** Jin Zhao, Ting Jia, Xiuming Wang, Yiming Xiao, Xingqu Wu

**Affiliations:** ^1^School of Education, Guangzhou University, Guangzhou, China; ^2^Department of Psychiatry, 987th Hospital of PLA, Baoji, China; ^3^School of Economics and Statistics, Guangzhou University, Guangzhou, China

**Keywords:** social media addiction, impulsivity, self-esteem, emotion, attentional bias

## Abstract

The use of social media is becoming a necessary daily activity in today’s society. Excessive and compulsive use of social media may lead to social media addiction (SMA). The main aim of this study was to investigate whether demographic factors (including age and gender), impulsivity, self-esteem, emotions, and attentional bias were risk factors associated with SMA. The study was conducted in a non-clinical sample of college students (*N* = 520), ranging in age from 16 to 23 years, including 277 females (53%) and 243 males (47%). All participants completed a survey measuring impulsivity, self-esteem, anxiety, depression, social anxiety, loneliness, and attentional bias. The final hierarchical regression model indicated significant risk factors for SMA with an accuracy of 38%. The identified set of associated risk factors included female gender (β = −0.21, *t* = −4.88, *p* < 0.001), impulsivity (β = 0.34, *t* = 8.50, *p* < 0.001), self-esteem (β = −0.20, *t* = −4.38, *p* < 0.001), anxiety (β = 0.24, *t* = 4.43, *p* < 0.001), social anxiety (β = 0.25, *t* = 5.79, *p* < 0.001), and negative attentional biases (β = 0.31, *t* = 8.01, *p* < 0.001). Finally, a discussion of the results is presented, followed by corresponding recommendations for future studies.

## Introduction

Social media (e.g., Facebook, WeChat, Tik Tok) have attracted substantial public interest to the point that they are becoming a cornerstone of modern communication. It has been argued that social media promote social interaction, help in maintaining relationships, and allow for self-expression (Baccarella et al., 2018). According to a survey by the China Internet Network Information Center, there are 900 million users of social media in China. College students are freer than others to control the use of their time and the use of social media is thus becoming an integral part of their lives. However, social media, if used immoderately, may lead to social media addiction (SMA), which refers to the excessive and compulsive use of social media platforms, resulting in severe impairment in all aspects of life (Kuss and Griffiths, 2017). Addicted users of social media tend to spend too much time on social media, to be overly concerned about social media and to be driven by uncontrollable urges to use social media (Andreassen and Pallesen, 2014). SMA can be viewed as a specific form of digital technology addiction, in which the conceptualizations all center on these addictive behaviors as pathological forms of necessary and normal behaviors (Moreno et al., 2021). SMA may affect users’ mental health, leading to anxiety, depression, lower subjective wellbeing, and poor academic performance (Lin et al., 2016). The present study will examine potential risk factors associated with SMA focusing on demographic factors, impulsivity, self-esteem, emotions, and attentional bias.

In general, the impact of demographic factors such as age and gender has been considered in previous studies. Young individuals maintain an online presence and develop addictive behaviors more often than older individuals (Abbasi, 2019). Furthermore, women are more likely to indulge in social media more than men in order to enhance their social connections (Andreassen et al., 2017).

Impulsivity is an important personality trait that plays a major part in the occurrence, development, and maintenance of addiction (Cerniglia et al., 2019). However, the link between impulsivity and SMA is controversial. It has been found that trait impulsivity is a marker for vulnerability to SMA (Sindermann et al., 2020). The most influential theoretical explanation for this is Dual System Theory, which is also known as reflective–impulsive theory. The reflective system includes the prefrontal cortex, which plays a key role in a wide range of executive and inhibitory behaviors, such as short-term memory, planning, attention, and resistance to immediate rewards for the sake of long-term rewards. By contrast, the impulsive system includes the subcortical brain areas, accounts for pleasure and addictive behaviors, and responds to quickly acquired cues regardless of long-term negative results. Imbalance between the reflective and impulsive systems leads to addictive behaviors (Droutman et al., 2019). However, another empirical study based on a Go/Stop Impulsivity task found impulsivity was not significantly associated with SMA (Chung et al., 2019). This inconsistency of results may be caused by the use of different measurement approaches. Therefore, the association between impulsivity and SMA needs further exploration.

Self-esteem impacts the predisposition to SMA and there is a negative association between the frequency of Facebook use, the meaning attributed to Facebook use, and users’ levels of self-esteem (Błachnio et al., 2016). People with low levels of self-esteem prefer to avoid face-to-face communication and escape into the virtual world where they can behave anonymously and do what they want. Also, negative feedback from social media will reduce users’ levels of self-esteem (Andreassen et al., 2017).

Concerns over the negative emotions of social media addicts have not abated. Prior studies have mainly considered the influence of anxiety, depression, social anxiety, and loneliness on SMA. Atroszko et al. (2018) reported that SMA is positively associated with anxiety and depression. Additionally, social anxiety and loneliness are the emotions generated in the process of interpersonal communication (O’Day et al., 2019). People with social anxiety prefer online communication as a way to avoid uncomfortable real interactions and social tensions. Caplan (2007) used privacy to explain this phenomenon: privacy can be better protected through online communication. However, the relationship between SMA and loneliness is controversial. Primack et al. (2017) regarded loneliness as a risk factor associated with SMA, indicating that high levels of loneliness may lead to addiction. Another study by Baltacı (2019) suggested that loneliness was not significantly associated with SMA. Thus, more studies examining the links between loneliness and SMA are needed.

In terms of cognitive factors, attentional bias has been considered as a potential causative factor of SMA. Attentional bias refers to a situation in which individuals are highly sensitive and allocate attentional resources to specific stimuli (Gao et al., 2011). Generally, substance and behavioral addicts display an attentional bias mainly toward negative information (Hu et al., 2020). Furthermore, attention to negative information (ANI) may further aggravate addictive behaviors (Cheetham et al., 2010). An important theoretical explanation for addicts’ ANI is the self-schema theory (Becker and Leinenger, 2011). Schemas are relatively stable and lasting cognitive templates for individual storage, organization, integration and information processing. A negative schema will make individuals pay attention to information consistent with the schema, resulting in a processing bias. It is not yet clear whether the attentional bias effect generalizes to SMA as a specific form of digital technology addiction. To our knowledge, no studies have specifically revealed a relationship between ANI and SMA.

Prior studies have focused on only one or two independent factors without considering the hierarchical importance of risk variables. Undoubtedly, identifying the hierarchical importance of risks has implications for the treatment and intervention of SMA. In the present study, an attempt was made to explore the risk factors for SMA considering their hierarchical importance. Also, this is the first report to specifically look at ANI and SMA with self-reported questionnaires, which provide the advantages of saving time and the capability to conduct large-scale investigations. It was hypothesized that each variable would be a significant predictor for SMA at each step.

## Materials and Methods

### Participants

A total of 532 college students attending a state university in China participated in the present study. 12 participants did not meet the inclusion criteria and were excluded. The final study sample consisted of 520 participants including 277 females (53%) and 243 males (47%). The ages of all participants ranged from 16 to 23 years (*M* = 19.68, *SD* = 1.07). Inclusion criteria for participants included fluency in Chinese and having at least one social media application account. Exclusion criteria included current psychiatric conditions and a history of mental illness (e.g., anxiety, depression, schizophrenia or bipolar disorder), as well as other addictive behaviors or a family history of addictions (e.g., alcohol use disorder, nicotine abuse, illegal drug dependence, etc.).

### Procedure

All participants completed paper-and-pencil surveys in class. Written consent was obtained from the participants before the survey. The survey took approximately 20 min. Data collection took place from April to June 2021.

### Ethics Statement

Approval for the research was granted by the ethics committee of Guangzhou University. All participants were informed of the purpose and procedures of the study, and that participation was anonymous and voluntary.

### Measures

Socio-demographics information: The survey recorded questions concerning age, gender, presence of social media accounts, current and prior of mental illness, as well as the presence of other addictive behaviors and a family history of addiction.

#### Bergen Social Media Addiction Scale

The Chinese version (Leung et al., 2020), adapted from Andreassen et al. (2017), was used to evaluate levels of SMA with higher scores indicating greater SMA. It consists of six items (e.g., “How often have you felt an urge to use social media more and more during the last year?”) measured on a 5-point scale (1 = *very rarely*, 5 = *very often*). According to the gold standard of clinical diagnosis, a BSMAS score of 24 was taken to be the optimal cut-off point (Luo et al., 2021). If the BSMAS score was 24 or above, the participant was considered to be addicted. Otherwise, the participant was considered to be non-addicted. The Cronbach’s alpha was 0.78 in the current study.

#### Brief Barratt Impulsivity Scale

The Chinese version (Luo et al., 2020), adapted from Morean et al. (2014), was used to measure trait impulsivity. It consists of eight items (e.g., “I do things without consideration”) rated on a 4-point scale (1 = *very inconsistent*, 4 = *very consistent*). Higher scores indicate poor self-regulation and impulsive behaviors. The Cronbach’s alpha was 0.81 in the current study.

#### Rosenberg Self-Esteem Scale

The Chinese version (Wang et al., 2010), adapted from Rosenberg (1965), was used to evaluate levels of self-esteem. It is rated on a 4-point scale (1 = *strongly disagree*, 4 = *strongly agree*) with 10 items (e.g., “I feel that I have a number of good qualities”). Higher scores indicate higher levels of self-esteem. The Cronbach’s alpha was 0.91 in the current study.

#### Self-Rating Anxiety Scale

The Chinese version (Liu et al., 1995), adapted from Zung (1971), was used to measure anxiety. It is rated on a 4-point scale (1 = *never or very rarely*, 4 = *most or all of the time*) with 20 items (e.g., “I feel more nervous and anxious than usual”). Higher scores indicate more severe anxiety symptoms. The Cronbach’s alpha was 0.84 in the current study.

#### Self-Rating Depression Scale

The Chinese version (Liu et al., 1994), adapted from Zung (1965), was used to assess depression. It consists of 20 items (e.g., “I feel gloomy and depressed”) rated on a 4-point scale (1 = *never or rarely*, 4 = *most or all of the time*). Higher scores indicate more severe depressive symptoms. The Cronbach’s alpha was 0.85 in the current study.

#### Interaction Anxiety Scale

The Chinese version (Peng et al., 2004), adapted from Leary (1983), was used to assess social anxiety. It consists of 15 items (e.g., “I will be nervous during an interview”) rated on a 5-point scale (1 = *not at all consistent*, 5 = *extremely consistent*) where higher scores represent greater social anxiety. The Cronbach’s alpha was 0.88 in the current study.

#### UCLA Loneliness Scale

The Chinese version (Liu, 1999), adapted from Russell (1996), was used to assess loneliness. It is composed of 20 items (e.g., “Do you often feel that no one can be trusted?”) rated on a 4-point scale (1 = *never*, 4 = *always*). Higher scores indicate higher levels of loneliness. The Cronbach’s alpha was 0.91 in the current study.

#### Attention to Positive and Negative Inventory

The Chinese version (Dai et al., 2015), adapted from Noguchi et al. (2006), was used to assess attentional bias. The inventory is composed of 22 items rated on a 5-point scale (1 = *totally inconsistent*, 5 = *totally consistent*) and includes two dimensions: attention to positive information with 12 items and ANI with 10 items (e.g., “I can’t forget the harm that others have done to me”). This study focused on the impact of ANI on SMA, thus only the ANI subscale was used. Higher scores on the ANI subscale indicate greater ANI. The Cronbach’s alpha was 0.73 in the current study.

### Data Analysis

Data were analyzed using the SPSS 24.0 software package program. Initially, the effects of demographic information (age and gender) on SMA in the total sample were checked with one-way ANOVAs. The Pearson correlation coefficient was conducted to reveal the links between gender, impulsivity, self-esteem, emotions, attentional biases and SMA. Finally, a hierarchical regression analysis was used to explore whether independent variables (i.e., gender, impulsivity, self-esteem, emotions, and attentional biases) could predict the dependent variable (SMA).

## Results

### Descriptive Data and Inter-Correlations Between Variables

First, one-way ANOVAs were used to investigate effects of age and gender on SMA in the total sample. Univariate analyses indicated that there is no significant difference by age [*F*_(7, 512)_ = 1.74, *p* = 0.09] but that the samples differed by gender [*F*_(1, 518)_ = 23.79, *p* < 0.001]. Females are more likely than males to be addicted to social media. Thus, the first step was to control for the effects of gender in the regression analyses. Next, a correlation analysis was performed on the influencing factors of SMA in the total sample. Bivariate correlations between variables are presented in Table 1.

**TABLE 1:** Descriptive data and inter-correlations between variables.

Variables	M (SD)	1	2	3	4	6	5	7	8	9	10
1. Age	19.68 (1.07)	1	0.04	–0.05	0.02	–0.07	–0.01	0.02	–0.07	0.02	0.06
2. Gender ^a^	0.47 (0.53)		1	0.01	0.05	–0.08	–0.05	−0.22***	–0.02	–0.04	−0.21***
3. Impulsivity	17.31 (4.03)		1	−0.48***	0.45***	0.31***	0.30***	0.46***	0.15**	0.34***	
4. Self-esteem	29.27 (4.73)			1	−0.61***	−0.43***	−0.41***	−0.58***	−0.29***	−0.33***	
5. Anxiety	42.24 (8.60)				0.74***	1	0.29***	0.51***	0.27***	0.41***	
6. Depression	46.44 (9.37)				1		0.38***	0.64***	0.30***	0.41***	
7. Social Anxiety	46.91 (9.96)						1	0.42***	0.38***	0.42***	
8. Loneliness	41.88 (9.33)							1	0.33***	0.33***	
9. ANI	34.54 (5.13)								1	0.45***	
10. SMA	16.03 (4.12)									1	

N = 520. **p < 0.01 and ***p < 0.001.

*^a^Dummy variable is coded as male = 1, female = 0. The proportion of females in the sample is 53%.*

*ANI, attention to negative information; SMA, social media addiction.*

### Hierarchical Regressions

Hierarchical regressions are presented in Table 2. Gender was included in Step 1 (*R*^2^ = 0.04). It was found that gender was significantly related to SMA (β = −0.21, *t* = −4.88, *p* < 0.001) and that females are more prone to addictive use of social media. In Step 2, gender and impulsivity remained risk factors (*R*^2^ = 0.16). Impulsivity was positively associated with SMA (β = 0.34, *t* = 8.50, *p* < 0.001). In Step 3, gender, impulsivity and self-esteem were included (*R*^2^ = 0.19). A higher level of self-esteem proved to be a protective factor associated with SMA (β = −0.20, *t* = −4.38, *p* < 0.001). Gender and impulsivity were still risk factors. In Step 4, negative emotions were added to the model (*R*^2^ = 0.30) and risk factors associated with SMA were found to include gender (β = −0.14, *t* = −3.79, *p* < 0.001), impulsivity (β = 0.18, *t* = 4.01, *p* < 0.001), anxiety (β = 0.24, *t* = 4.43, *p* < 0.001), and social anxiety (β = 0.25, *t* = 5.79, *p* < 0.001). Depression, loneliness and self-esteem were not risk factors. In Step 5, ANI was shown to be positively correlated with SMA (β = 0.31, *t* = 8.01, *p* < 0.001). The final model accounted for 38% of the variance [*F*_(9, 510)_ = 36.61, *p* < 0.001]. In the final model, gender, impulsivity, anxiety, social anxiety, and ANI were all found to be risk factors associated with SMA.

**TABLE 2:** Regression analyses.

Steps	Variables	β	*t*	*p*
Step 1	Gender^a^	−0.21	−4.88	<0.001
	*R*^2^ = 0.04, *F*_(1, 518)_ = 23.79***			
Step 2	Gender^a^	−0.21	−5.27	<0.001
	Impulsivity	0.34	8.50	<0.001
	*R*^2^ = 0.16, Δ*R*^2^ = 0.12, *F*_(2, 517)_ = 34.66***			
Step 3	Gender^a^	−0.20	−5.09	<0.001
	Impulsivity	0.25	5.50	<0.001
	Self-esteem	−0.20	−4.38	<0.001
	*R*^2^ = 0.19, Δ*R*^2^ = 0.03, *F*_(3, 516)_ = 40.64***			
Step 4	Gender^a^	−0.14	−3.79	<0.001
	Impulsivity	0.18	4.01	<0.001
	Self-esteem	−0.01	−0.10	0.92
	Anxiety	0.24	4.43	<0.001
	Depression	0.07	0.98	0.33
	Social anxiety	0.25	5.79	<0.001
	Loneliness	−0.03	−0.54	0.59
	*R*^2^ = 0.30, Δ*R*^2^ = 0.11, *F*_(7, 512)_ = 33.07***			
Step 5	Gender^a^	−0.15	−4.25	<0.001
	Impulsivity	0.20	4.78	<0.001
	Self-esteem	0.02	0.31	0.76
	Anxiety	0.21	4.13	<0.001
	Depression	0.05	0.87	0.39
	Social anxiety	0.16	3.83	<0.001
	Loneliness	−0.07	−1.41	0.16
	ANI	0.31	8.01	<0.001
	*R*^2^ = 0.38, Δ*R*^2^ = 0.08, *F*_(8, 511)_ = 40.54***			

N = 520. ***p < 0.001.

*^a^Dummy variable is coded as male = 1, female = 0.*

*ANI, attention to negative information.*

## Discussion

The main objective of this study was to examine whether demographic factors, impulsivity, self-esteem, emotions, and attentional biases were potential risk factors associated with SMA. It was found that females were more susceptible to SMA than males. Additionally, impulsivity, low levels of self-esteem, anxiety, social anxiety, and ANI were found to be risk factors for SMA.

### Demographic Factors

Gender was found to be associated with SMA. In the present sample, 2.9% of the participants scored 24 or above in BSMAS and thus met the criteria for SMA. The proportion was similar to the previous report (3.5%) in a Chinese sample (Luo et al., 2021). The prevalence of SMA in males and females in the current study was 1.2 and 4.3%, respectively. Females showed higher addiction rate and greater levels of SMA than males. This result is in agreement with prior research (Monacis et al., 2020). Females focus more attention on social activities for enhancing communication and prefer to share more selfies on social applications and social networking sites (Dhir et al., 2016). Interestingly, it was found that age had no significant effect on SMA. This finding is inconsistent with the prior study that young people are more likely to develop SMA (Abbasi, 2019). The lack of association between age and SMA can possibly be attributed to the selected sample in which participants were relatively young and the age span was small, resulting in no age effect.

### Impulsivity

Although, the association between impulsivity and SMA is still controversial, this study supports the hypothesis that impulsivity is positively associated with SMA. This finding is in agreement with the study by Sindermann et al. (2020), which indicated that trait impulsivity was positively associated with the severity of SMA. It contradicts the study by Chung et al. (2019), which indicated that impulsivity was not associated with SMA. Our finding underlines the importance of impulsivity as a risk factor related with SMA. This result may be supported by Dual System Theory (Droutman et al., 2019). Higher levels of impulsivity in social media addicts are rooted in an imbalance between the reflective and impulsive systems. Higher levels of impulsivity might be associated with SMA due to attentional fluctuation, i.e., individuals engage in social media when they lose attention to another task. Addictive uses of social media can thus be regarded as a form of urgency relevant behaviors displayed to regulate (suppress and/or exacerbate) emotional states in the short term despite the delayed negative consequences (Rothen et al., 2018). Similarly, a study by Minhas et al. (2021) explored the links between alcohol abuse and food addiction in relation to impulsive personality traits, impulsive choices and impulsive action. It was found that alcohol problems and food addiction showed parallel associations, indicating common underlying impulsivity mechanisms. Likewise, the present study also found that a higher level of impulsivity is a risk factor for SMA. Collectively, the multiple lines of evidence suggest that SMA, food addiction, and alcohol abuse may have similar underlying impulsivity mechanisms.

### Self-Esteem

Levels of self-esteem were found to be negatively correlated with SMA in the current study. This is consistent with a prior study that found higher levels of self-esteem are a protective factor against addictive behaviors (Andreassen et al., 2017). In the research on Internet addiction, people with low levels of self-esteem tend to use the Internet for social support, and the social support gained from the Internet could compensate for the lack of social support offline. Also, SMA showed a negative correlation with levels of self-esteem. Individuals use more social media to obtain higher levels of self-esteem (e.g., harvesting “likes”), and/or to get rid of feelings of low self-esteem (Błachnio et al., 2016). Notably, after emotions were incorporated into the model, self-esteem was no longer a risk factor for SMA in hierarchical regressions. Consistent with prior research, this suggests that the influence of self-esteem on SMA is regulated by emotions (Andreassen et al., 2017).

### Emotions

The results of this study show that emotions, particularly, anxiety and social anxiety, are the strongest risk factors associated with SMA. This is consistent with prior research showing that anxiety is a risk factor for SMA (Keles et al., 2019). Anxious individuals prefer to use social media platforms to alleviate unfavorable emotions, for example, by seeking attention, support, or a sense of belonging on social media (Vannucci et al., 2017). Additionally, in line with the study by Baltacı (2019), this study found that social anxiety is positively associated with SMA. Individuals who experience difficulty communicating with others in social environments prefer social media for interaction. Privacy is an important feature of the Internet (Caplan, 2007). Compared to face-to-face communication, interaction through a virtual environment is perceived to be less risky.

The relationships between depression, loneliness, and SMA were found to be relatively low. Neither loneliness nor depression was significantly associated with SMA in this study. This is consistent with prior research that has shown that depression and loneliness were not predictors of SMA (Baltacı, 2019; Marttila et al., 2021). The reason for the lack of a link may be the marginal effect caused by these moderate relationships. When depression and loneliness were analyzed as psychosocial variables in terms of SMA, it was found that depression and loneliness are both the reasons for Primack et al. (2017) and the consequences of SMA (Dossey, 2014).

### Attention to Negative Information

This was the first study to look at the association between ANI and SMA. This study used hierarchical regressions to find that ANI is one of the risk factors associated with SMA. Previously, a study by Aguilar de Arcos et al. (2008) reported that opioid users have higher arousal responses to negative and unpleasant emotional images compared with healthy individuals. Similarly, another study by Hu et al. (2020) used eye tracking technology to find that mobile phone addicts show a processing bias toward negative emotional clues. Although, unlike substance and behavioral addiction, the availability of social media is so high. Social media addicts also displayed negative attentional bias effect. This indicates that a processing bias toward negative information may be the common underlying mechanism that incurs and maintains addictive behavior. The abovementioned phenomenon can be explained by self-schema theory (Becker and Leinenger, 2011). Addicts mainly demonstrate attentional biases toward negative emotions because they often experience negative emotions such as anxiety and depression. Information consistent with the negative schema will automatically capture the individual’s attention, leading to negative attentional bias. ANI is also an important reason for the occurrence, development, and maintenance of social anxiety and depression (Brailovskaia and Margraf, 2020). Individuals with high levels of social anxiety specifically allocate attentional resources to negative information in the environment and social interactions, resulting in depression.

### Implications

Our study can not only provide theoretical and practical support for prevention and intervention into SMA, but also contribute to improving individuals’ physical and mental health. It was found that female gender, impulsivity, self-esteem, anxiety, social anxiety, and ANI exhibited significant risk effects for SMA. In future studies, alleviating users’ anxiety, actively organizing social activities, and correcting attentional bias with attention training programs could be used to reduce the risk of SMA.

## Limitations and Future Directions

The current study has some limitations. First, since this research was based on a single classroom survey, it was a relatively small study in terms of scope, and the sample was potentially unrepresentative. Second, less information was collected from the participants in the demographic characteristics portion of the survey, resulting in a lack of some sociodemographic and clinical information about participants. Third, data were collected near the end of the semester, when senior students were preparing for internship and/or employment. Thus, participants mainly belonged to junior grades. The age span is relatively narrow, which may have affected our ability to detect an effect of age on SMA. Fourth, this research was based on questionnaires and was limited by self-report measurement methods. The validity of the research may depend on the accuracy of participants’ reports. Finally, as this study was cross-sectional design, the causality between variables could not be determined.

In future studies, the scope of sampling can be further expanded to enhance the representativeness of the sample and explore the effect of age on SMA. Also, a wide range of other information about participants should be gathered through the survey to explore the effect of demographic characteristics on SMA: e.g., average daily time spent on social media, the number of social media applications, discipline background, etc. Moreover, research focused on the relationship between impulsivity, attentional biases and SMA could be combined with empirical research. For example, the Go/Stop paradigm and Stroop task could be used to assess impulsive action and attentional bias, respectively. Finally, longitudinal tracking research could be used to determine the causality between variables.

## Data Availability Statement

The original contributions presented in the study are included in the article/supplementary material, further inquiries can be directed to the corresponding author.

## Ethics Statement

The studies involving human participants were reviewed and approved by the Ethics Committee of Guangzhou University. Participants provided written informed consent to participate in the study.

## Author Contributions

JZ designed the project and collected the data. TJ, XWa, and YX conducted statistical analyses. XWu was involved in supervision and edit manuscript drafts. All authors approved the final manuscript before submission.

## Conflict of Interest

The authors declare that the research was conducted in the absence of any commercial or financial relationships that could be construed as a potential conflict of interest.

## Publisher’s Note

All claims expressed in this article are solely those of the authors and do not necessarily represent those of their affiliated organizations, or those of the publisher, the editors and the reviewers. Any product that may be evaluated in this article, or claim that may be made by its manufacturer, is not guaranteed or endorsed by the publisher.
